# Biomedical career enrichment programs: Exploring women and minority participants’ motivators and outcomes

**DOI:** 10.1371/journal.pone.0228934

**Published:** 2020-02-14

**Authors:** Rishita Bhatt, Bernadette West, Sunita Chaudhary

**Affiliations:** 1 School of Public Health, Rutgers, The State University of New Jersey, Piscataway, NJ, United States of America; 2 Department of Surgical Oncology, Rutgers Cancer Institute of New Jersey Rutgers, The State University of New Jersey, New Brunswick, NJ, United States of America; Charles P. Darby Children's Research Institute, UNITED STATES

## Abstract

Limited empirical data exists on why women and minority students enter Biomedical Career Enrichment Programs (BCEPs) and how program variables—such as duration of research—influence their intention to pursue research careers. This exploratory study reports motivators for participation in BCEPs among women and racial/ethnic minority students—historically underrepresented groups—and the influence of program and personal variables on their research-career intent and self-efficacy beliefs. We studied the program variables of research experience, research duration, and mentor influence; and the personal variables of race, gender, family, and peers. Using the conceptual framework of planned behavior theory and social cognitive career theory, we interviewed students from underrepresented groups participating in BCEPs that offered research experience for short duration (Group A), long duration (Group B), and no research experience (Group C). We utilized Atlas Ti, a qualitative methodological software tool, to analyze the interview responses. Students choosing a BCEP with research experience cited “opportunity to gain experience” and “interest or curiosity in research” as motivators. Duration of research experience had a positive relationship with enhancement in research skills and self-efficacy beliefs, but did not change the initial research-career intent of these BCEP participants. The study revealed an interesting and unexpected theme of “perceived deterrents” to a career in research that included stress of competition (e.g. grants), the instability of projects, and the isolation of scientific research. Importantly, the study findings indicate the need to reform program design and science policies that challenge the current biomedical workforce and dissuade interested students from underrepresented groups from entering the field.

## Introduction

Representation of women and racial and ethnic minorities remains low in the biomedical workforce [1]. Increasing diversity in the biomedical field is important for reducing disparities in health care access, promoting health disparities research, and developing thought leaders in the field [1, 2]. To motivate and retain talented professionals from underrepresented groups (URGs) in the biomedical workforce, there is a need to address a multitude of interrelated issues that diminish the academic, financial, social, and cultural capital available to students from URGs [3, 4].

Biomedical Career Enrichment Programs (BCEPs) have been utilized as a “pipeline” strategy to prepare, motivate, and support URG youth for entry and retention—initially in the broader STEM careers—and ultimately in the biomedical workforce [4, 5, 6]. A number of studies show that undergraduate students participating in enrichment programs—especially those that include research experience—report enhanced academic skills, self-efficacy beliefs, and positive attitudes (motivation and intent) for entry and persistence in STEM fields [6, 7, 8].

Although several studies have documented positive outcomes from research experiences and shown benefits to the development of various skills, scant empirical data is available on processes and factors that motivate students to make the *initial choice* to participate in research-experience-based BCEPs. Our exploratory qualitative study asks the following two research questions: 1) *why* students from underrepresented groups choose to participate in research experiences; and 2) *which* personal and program-related variables influence the participants’ attitudes and research career choices *longitudinally* over the duration of their exposure to formalized research experience in BCEP in our specific program and institutional context. A qualitative research approach using interviews provides a more nuanced picture of the processes underlying the educational and career choices of individuals with different social identities. Better understanding of the modulating effect of participants’ personal/demographic variables (e.g. gender, race/ethnicity, influence of family and peers) and the role of program variables (e.g. duration, mentors) could enable more effective targeting of current limited resources, as well as assist in building stronger support systems for underrepresented students.

### Research participation during high school

Benefits of research experiences have been reported as early as high school level for students from URGs participating in enrichment programs [9]. Enrichment programs providing summer research internships engage URG high school students early on during their academic trajectories, so they can continue to learn and benefit from the research opportunities available in college [10, 11, 12, 13]. Oseguera et al. [14] reported that earlier academic exposure to scientific research (through participation in enrichment programs) in high school is positively correlated with undergraduate students’ intention to pursue scientific research careers and probability of entry into a STEM field. A study by Rohrbaugh and Corces [15] showed that URG students participating in research experiences during high school have a higher GPA, improved critical thinking skills, and increased motivation to pursue science majors and continued involvement in biomedical research in college. Participating in research experiences at the high school level and continuing throughout the academic trajectory might also be more effective in mitigating the lack of personal and academic resources that can discourage minority student’s entry or success in STEM fields. Thus, many institutions have endeavored to include high school and undergraduate level students in BCEPs [9, 16].

### Benefits of participation in research during undergraduate

In a study using Survey of Undergraduate Research Experience to evaluate the benefits of summer research for undergraduate students, the majority of students reported enhanced or sustained intent to remain in a science field. Participants also expressed positive influence on self-reported measures of student learning of research skills such as “understanding of the research process in your field” [17]. In the same study, higher ratings of 13 learning measures (e.g. “readiness for more demanding research” and “tolerance for obstacles”) were associated with continued interest in the science field. In a study of alumni of a minority undergraduate biology enrichment program, Jones, Barlow, and Villarejo [5] reported participation in research had a positive influence on the decision to pursue a Ph.D. in biomedical research. Participation in research has been shown to increase understanding of the culture of scientific research and the day-to-day realities of a researcher [18], communication skills [19], and identity as a scientist [7, 20, 21] among undergraduates.

### Variables influencing outcomes of research experiences for minority and female students

While prior studies show the benefits of early exposure to research through intervention programs [17, 22], the advantages vary for minorities since they are less likely to participate in such research-related experiences [23]. Fewer black, Latino, and first-generation college students have engaged in research as undergraduates, as compared to white and Asian students [23]. This study further showed that deterrents to participation in undergraduate research included lack of academic preparation, knowledge, and/or understanding of the value of research opportunities, as well as off-campus work. [23]. Another study showed that undergraduate minority students’ participation in science research interventions is influenced by their overall college experience, social background characteristics, and whether the institutional environment presents easy access or barriers to the research programs [24].

Program variables intrinsic to the research experience itself, such as the mentor-mentee relationship, nature of the project, and duration of research, also influence student outcomes [25]. In addition to program variables, several personal variables, such as support from family and peers, race/ethnicity, and gender, might modulate the benefits of research experience for youth from URGs.

Research has shown that parental support constitutes most of the cultural and social capital available to the student and plays a critical role in participation in STEM activities during early learning experiences [26, 27]. For example, Mexican American high school students’ perception of high levels of family support to pursue a math/science career and related educational choices directly influences their math/science goals, mediated through their self-efficacy beliefs [28]. Fouad, et al. [29] reported that college women identified parents’ lack of knowledge and support as a barrier for persistence in STEM. The role of parental support at later academic levels in the career decision-making process is equivocal. McCallum [30] explored the role of parents in the career decision-making process of African American doctoral students and found that, by providing “insights, resources, emotional, and social support,” parents play an important role in entry and retention in graduate school. However, other studies have shown that family does not appear to influence career decisions later in life [31].

Gender is another demographic variable that is likely to influence the outcomes of research experience during enrichment programs. Using pre- and post-surveys, Kardash [32] evaluated gender differences in growth of research skills of undergraduate student participants in enrichment programs. The study reported that while no gender differences in research skill were observed in the pre-survey, women participants reported lower increases in research skills such as “ability to understand concepts in the field” and “ability to formulate research hypothesis” following participation in the program, compared to those reported by male participants. In another study, women participants of undergraduate research programs had higher increases in self-efficacy beliefs, as well as interest in science when compared to men, and they attributed participation in research programs as the main reason for pursuing graduate school [33]. Aikens, et al. [34] reported that women participants of undergraduate research experiences showed less identity as a scientist, scholarly productivity, and intention to pursue STEM Ph.D.s as compared to men, although this was mediated partly through the mentoring relationships of women with their faculty advisors.

Positive mentoring relationships during high school and undergraduate studies encourage minority students to pursue STEM and science research careers [35]. Haeger and Fresquez [36] found that within the context of research experience, mentoring undergraduate minority students resulted in academic gains such as increased cumulative GPA compared to the control group. Studies have shown that effective mentoring relationships affect positive changes in research self-efficacy beliefs, the sense of belonging to the scientific community, and identity as a scientist; which result in motivation, persistence, and commitment to science research careers [8, 37]. Mentoring support becomes more critical for students from underrepresented groups facing significant barriers to entry and persistence in STEM fields. There is some evidence that undergraduate students from underrepresented backgrounds benefit from longer-term mentoring relationships in the context of their research experience, which then lead students to self-report higher gains in “research skills, independence, and understanding of the research process” [36].

Evidence regarding the role of race/ethnicity in influencing the career outcomes of research experiences is also equivocal. Oseguera, et al. [14] examined the characteristics and experiences of entry-level freshman minority undergraduate students interested in biomedical and behavioral sciences. They reported that among American Indians, higher academic self-efficacy increased the likelihood of aspiring to a research career, while in the African American group there was no similar correlation. A study by Lopatto [17] reported higher learning gains in students from underrepresented groups following participation in a summer undergraduate research program, but the research experience had no effect on intention to pursue a postgraduate degree. In a larger survey study by Russell, et al. [18] examining the influence of undergraduate research on intent to pursue research careers, relatively small racial/ethnic group differences were reported in 15,000 respondents who participated in undergraduate research experiences.

Studies have demonstrated the benefits of longer duration of research training on several attributes critical for entry and retention in STEM fields. For example, higher percentages of undergraduate researchers with more than a year of research experience self-reported expectations of obtaining a Ph.D. compared to those with one-to-three-months of research experience [18]. Other studies demonstrated increased GPA, perceived benefits, enhancement of ability to carry out research [38], and research skill enhancement [39] in students who participated in science research programs for longer durations. Thiry et al. [8] showed gains in personality traits such as “patience, independence, and initiative,” and strengthening of identity as a scientist in undergraduate students participating in multi-year research experiences. Undergraduate research participation for a longer time (three terms or more) also increases graduation rates [5, 8, 40].

### Theoretical framework

Two specific theories guided the development of the interview process used in this study. The first, theory of planned behavior, states that an individual’s intention towards a planned behavior is shaped by positive or negative feelings toward the behavior, subjective norms about performance of the behavior, and the individual’s perception of the ease with which the behavior can be performed [41, 42]. The second theory, social cognitive career theory, states that three personal variables influence an individual’s career decisions: self-efficacy—one’s belief in their capability to perform an activity and take action to achieve a designated level of performance; intent to pursue a research career; and goals [43, 44, 45, 46].

Both aforementioned theories share several common themes.

1. The interaction of intrinsic person-related factors and extrinsic influences of the person’s environment shape career-related behavior.
2. Individuals’ beliefs about themselves play an important role in the decision-making process.
3. Perceived barriers and supports in an individual’s environment—including institutional environment and “important others” such as parents, mentors, and teachers—continually influence the decision-making process.
4. Ethnicity, gender, and socio-economic status have a direct overarching influence on all factors.
5. Career choices such as academic majors are not static but in a dynamic flux.

Lent and Brown [47] proposed an extension of the social cognitive career theory to encompass behavioral strategies individuals adapt to self-manage their careers. Their career self-management model recognizes that individuals utilize various self-directed strategies proactively for successful career development and decision-making. Additionally, self-efficacy beliefs modulate career self-management adaptive behaviors. Lent and Brown point to the influence of “process efficacy,” defined as “the perceived ability to manage specific tasks necessary for career preparation, entry, adjustment, or change across diverse occupational paths.” St. Clair et al. [48] explored how process efficacy among people with biomedical Ph.D.s influences their career development and exploration activities. For undergraduate students, process efficacy might include goal setting, choice of academic majors, choosing to participate in internships, and research-based BCEPs for career exploration and preparation.

## Materials and methods

We used a multiple comparative case study design with each case bounded by the duration of research experience of the BCEP participant. The study site is a major northeastern research university, which ranks high in ethnic/racial diversity nationally. Hispanic/Latino students comprise ~13% of the undergraduate student population, while Black or African American students constitute 7.5% of the student population with 40% white. The university has a good balance of males to females with ~50% males and ~50% females. Rutgers Institutional Review Board approved the research study.

### Biomedical Career Enrichment Programs (BCEPs) in this study

BCEPs are designed to promote diversity at our university. Some BCEPs include high school students and serve as “pipeline” programs for the university. High school students are included as studies show that research experience during high school motivates students to pursue STEM fields and majors in college and subsequent years [9, 20]. We recruited participants from seven BCEPs for underrepresented students at the university (see Table 1 for characteristics of the programs). Participants self-identified to a racial /ethnic category and gender. Our interviewees included high school and undergraduate students including women, African Americans, Hispanics/Latinos, and black/students of color.

**Table 1:** Characteristics of STEM programs used for student recruitment.

Program	Duration	Research Experience	Formal Courses / Workshops/ Seminars	Scientific Writing Skills	Oral Presentation Skills	Poster Session	Other
**P1**	Summer and academic year	Yes	Yes	Yes	Yes	Yes	Journal club
**P2**	Summer	Yes	Yes	Yes	Yes	Yes	Journal club
**P3**	Summer	Yes	Yes	Yes	Yes	Yes	GRE preparation, application to graduate school
**P4**	Summer and academic year	Yes	Yes	Yes	Yes	Yes	
**P5**	Summer and academic year	Yes	Yes	Yes	Yes	Yes	
**P6**	Summer	Yes	Yes	Yes	Yes	Yes	MCAT preparation
**P7**	Summer and academic year	No	Yes	No	No	No	Career counselling

All seven BCEP programs promote biomedical career choice by providing career development activities, internships, and academic support in general STEM courses. The first six programs in Table 1 (P1 through P6) include mentor-supervised biomedical laboratory research as a major component, share faculty, have a didactic component (seminars, workshops, and lectures), have an oral/written presentation requirement, and have a goal to encourage women and minorities to pursue biomedical research careers. Participants in the P1 through P6 programs spend 85–90% of their time conducting biomedical laboratory research under the guidance of a faculty mentor (principal investigator) and a peer mentor who is either a senior graduate student or a post-doctoral fellow in that principal investigator’s laboratory. Trainees spend 10–15% of their time attending lectures, workshops, and career counseling. Faculty at the university overlap as mentors in P1 through P6. Participants of P1 through P6 are encouraged to pursue a STEM degree and a career in the biomedical field. Thus, programs P1 through P6 are very similar and career outcome expectations are broad, though research is a major component of the programs.

The last program (P7 in Table 1) provides academic enrichment, support, and counseling for currently underrepresented groups to encourage them to pursue biomedical careers, but does not have a laboratory research component. Participants receive individual academic planning, group sessions in math and science, academic advising, career guidance, and assistance in submitting applications to medical schools [16]. While P7 program does not include research experience, students matriculating in this program have the option to pursue research experiences. Group A included two students in P7 who pursued short-term research, and Group B included one student in P7 who pursued long-term research.

### Study participant demographics

Inclusion criteria for study participants included the following: first-time participant of a BCEP at the university; self-identification as African American, black, Hispanic, American Indian, Pacific Islander, or a woman; at least 16 years of age; enrolled in a high school or undergraduate degree program; reads and writes English; and gives informed consent.

We recruited participants by email invitation sent to students participating in BCEPs that provided short-term, long-term, and no research experience. Study participants were selected from the pool of students that responded to this initial email and volunteered to participate in the interviews. Participants in the study received informed consent statements and were assured confidentiality and anonymity. Forty-five students were recruited into three separate groups of 15 students each.

- **Group A (short-duration research):** students who participated in a BCEP with more than 120 but less than 400 hours, within one academic year. The 120–400 hour time intervals were chosen as typical summer immersions are of 3 to 10 weeks full time (~40 hours per week) duration.
- **Group B (long-duration research):** students who participated in a BCEP for a time period longer than a typical summer internship (more than 400 but less than 800 hours of research, accomplished over one-to-two academic years).
- **Group C (no research):** students who participated in a BCEP that offered no laboratory research experience, thus serving as a control group.

Each of our three groups consisted of both college and high school students.

- Group A: 62% college students, 38% high school students.
- Group B: consisted of 77% college students, 23% high school students.
- Group C: 94% college students, 6% high school.

In both Group A and Group B, all college students were science majors demonstrating early interest in the field and intent to pursue science-related careers. Table 2 provides demographic information on all study participants.

**Table 2:** Demographic information on study participants.

Group	Academic Level*	Male	Female	African-American /Black	Hispanic/Latino	Asian/Unknown^
**A**	6 HS 9UG	2	13	4	5	6 (Females)
**B**	3 HS 12 UG	0	15	3	5	7 (Females)
**C**	2 HS 13 UG	3	12	3	9	3 (Females)

*HS, High School student; UG = Undergraduate student

^ Participants who self-identified as Asian or Unknown were all females.

### Interviews

Qualitative data collection using interviews provides a more robust understanding of the processes that occur to produce a more accurate and nuanced picture that gives value to the views and experiences of both students and research scientists [49]. This study utilized in-depth qualitative interviews to explore what motivates students to participate in a BCEP and which personal and program variables influenced their decision to pursue a scientific research career.

The first author conducted the interviews. She was an MPH student under the guidance and mentorship of the second and third authors. The second author is a Ph.D. with interest and expertise in sociological research, outcome evaluation, and public policy, and the third author is a Ph.D. biomedical researcher with interest in outcome evaluation of interventions among underrepresented students.

The study included a socio-demographic questionnaire and an interview guide assessing (a) their general research-career intention, (b) their scientific research self-efficacy and overall program benefits, and (c) family, peer, and mentor influence on career intention. We selected the interview questions listed in Table 3 (Interview Guide) from a pre-existing parent study in which questions were adapted from literature, as well as from existing survey tools and scales [16].

**Table 3:** Interview guide.

THEORY	RATIONALE	INFORMATION	QUESTIONS:
**Theory 2b & 2c: Social Cognitive Career Theory**. Individual career decisions are influenced by three personal variables. These questions focus on the following: • ^**2b**^Intent to pursue a research career. • ^**2c**^Goals (determination to engage in a particular activity or affect a particular outcome).	These set of questions provide basic demographic information. Responses to these interview questions provide insight in understanding factors that influence participants of structured intervention programs to pursue scientific research career.(e.g. initial interest and prior experience).	DEMOGRAPHIC	**1. What is your major?** • Science • Non-Science **2. How did you select this major?** • General Interest • Family / Friend Influence • Trial & Error • Positive Experience • Interest in Research • Other: Please explain **3. Interest in scientific research (pre-program).** • Yes or No? **4. Did you have any prior research experience prior to this program?** • Yes or No? • If yes, was it positive? • Yes or No?
**1. Theory 1c: Theory of Planned Behavior**. Intention towards a planned behavior is shaped by: • ^**1c**^The individual’s perception of the ease with which the behavior can be performed. **2**. **Theory 2c: Social Cognitive Career Theory**. Individual career decisions are influenced by three personal variables. This question focuses on the following: • ^**2c**^Goals (determination to engage in a particular activity or affect a particular outcome).	These interview questions provide understanding of program characteristics such as duration and timing of intervention (high school vs. undergraduate) and how these characteristics can be utilized for more effective targeting of resources.	DEMOGRAPHIC	**1. What Program did you participate in–length/duration?.** (get description of each). a. P1 b. P2 c. P3 d. P4 e. P5 f. P6 g. P7 h. OTHER
**Theory 1a & 1c: Theory of Planned Behavior**. Intention towards a planned behavior is shaped by: • ^**1a**^Individual’s positive or negative feelings toward the behavior • ^**1c**^The individual’s perception of the ease with which the behavior can be performed **2. Theory 2b: Social Cognitive Career Theory**–Individual career decisions are influenced by three personal variables. This question focuses on the following: • ^**2b**^Intent to pursue a research career	Responses to these interview questions provided understanding of factors that influence participants of structured intervention programs to pursue scientific research careers.	GENERAL	**1. Why did you choose to participate in the program**: a. Interest in Research/Curiosity b. Financial (stipend for participation) c. Looks good on resume d. Opportunity to gain experience e. Peers f. Other:
**Theory 2b & 2c: Social Cognitive Career Theory**. Individual career decisions are influenced by three personal variables. These set of questions focus on the following: • ^**2b**^Intent to pursue a research career • ^**2c**^Goals (determination to engage in a particular activity or affect a particular outcome)	Responses to these interview questions provide understanding of factors that influence participants of structured intervention programs to pursue scientific research careers. In addition, the questions were designed to collect some preliminary data on the benefits and deterrents to each program. By asking these questions, we wanted to assess any existing shortfalls to the programs. This was expected to assist in identifying factors that influence (positively or negatively), participants of these structured intervention programs to pursue scientific research careers.	GENERAL PROGRAM BENEFITS CAREER INTENTIONS	**1. Feedback on the program:** • Would you recommend this program to your friends? • If yes or no, state minimum three reasons for doing so. • If you had one suggestion for a future program, what would it be? • Do you see yourself in a research or science related career in the future? • Yes or No? & Why? **2. Career Intentions prior to attending program.** • Were you planning on pursuing a career in research prior to the program? • If no, what did you want to do? **3. Career intentions post attending program.** • Did you change your career path as a result of attending this program? • If so–provide at least 3 reasons. • If not–provide at least 3 reasons. **4. What is the ultimate career you plan on pursuing now?**
**Theory 2: Social Cognitive Career Theor**y. Individual career decisions are influenced by three personal variables. This question focuses on the following: • ^**2c**^Goals (determination to engage in a particular activity or affect a particular outcome)	This question was crucial in the study because it explored program characteristics such as duration and timing of intervention and how these characteristics would enable more effective targeting of resources. Students were asked whether they felt the length of the program made a difference in their overall feelings towards research. A positive experience, despite length, can help to influence a student to pursue a career in research sciences. Probe questions included whether feelings towards research and the program, even their ultimate career plan, would have been the same if the program had been half the length.	INTENT TO PURSUE RESEARCH CAREER	1. **Did you feel the time provided was sufficient in helping you fulfill what you hoped to acquire from this program?** Students felt program was: • Too Long–Made up their mind in less time than what they were provided. • Too Short–Needed more time to make decision on whether they liked research. • Just Right–Perfect amount of time to make decision on whether they wanted to pursue a career in the sciences
**1. Theory 1a**: **Theory of Planned Behavior**. Intention towards a planned behavior is shaped by: • ^**1a**^Individual’s positive or negative feelings toward the behavior. **2. Theory 2b: Social Cognitive Career Theory**. Individual career decisions are influenced by three personal variables: • ^**2b**^Intent to pursue a research career	These set of questions were designed to measure student’s confidence level and general effectiveness in exposing students to the research based sciences. By asking about the student’s feelings towards the program and their confidence levels (before & after) we explored the pros and cons of the program and how that may also have influenced the student to choose a particular career path.	SELF-REPORTED EFFICACY (Post-Program)	**1. Self-reported competency rating.** • Do you feel confident that you would be able to determine what is or what is not valid scientific evidence? • If no–please explain. • Can you interpret data tables & graphs? • If no–please explain. • Do you feel that you could pose questions that can be addressed by collecting and evaluating scientific evidence? • If no–please explain. • Do you feel capable in writing a report using scientific data as evidence? • If no–please explain.
**Theory 1b: Theory of Planned Behavior**. Intention towards a planned behavior is shaped by: • ^**1b**^Subjective norms (expectations of other individuals important to the individual) about performance of the behavior	This set of questions was designed to give further insight into the role of family as well as peers, both very critical forces in shaping career choices of URM youth, which will allow for incorporation of strategies that include family and peers in intervention program design.	FAMILY & PEER	**1. Has anyone every encouraged you (or discouraged) you in pursuing a career in scientific research? If so, who & how?** • Family/Peer • Teacher • Lab instructor in the program? • Program

1. Theory of Planned Behavior–Intention towards a planned behavior is shaped by

1a. Individual’s positive or negative feelings toward the behavior

1b. Subjective norms (expectations of other individuals important to the individual) about performance of the behavior

1c. The individual’s perception of the ease with which the behavior can be performed

2. Social Cognitive Career Theory–Individual career decisions are influenced by three personal variables

2a. Self-efficacy (one’s belief in their capability to perform an activity and take action to achieve a designated level of performance)

2b. Intent to pursue a research career

2c. Goals (determination to engage in a particular activity or affect a particular outcome)

These interview questions were designed to provide supplementary insight to the already existing quantitative tool by aiding in furthering understanding:

1. Factors that influence participants of structured intervention programs to pursue scientific research careers
2. Program characteristics such as duration and timing of intervention (high school vs. undergraduate) and how these characteristics would enable more effective targeting of resources.
3. The role of family as well as peers, both very critical forces in shaping career choices of URG youth, which will allow for incorporation of strategies that include family and peers in intervention program design.

The Institutional Review Board of the University approved the study. Each participant received a consent form detailing the study objectives, duration of the interview, data confidentiality, and voluntary participation in interviews for the research study. Interviews were conducted in person, as well as over the telephone with 15 participants. The data being gathered started to become repetitive and saturation was reached with this sample size. Interviews were conducted for 45–60 minutes at specific locations at the university, following or preceding a scheduled program event to make it easier for participants. We audiotaped the interviews to record the responses accurately. The study participants received a $10.00 gift card at the completion of the interview as compensation for their time.

### Data analysis

Each interview was audio recorded and later transcribed into a Word document. The first and second authors developed a set of codes based on key variables of interest in the literature—as well as from careful reading and rereading of the transcripts for themes—and used it to code the unstructured data. (See Table 4: Coding Guideline.) Coding themes helped to elucidate the potential differences or similarities between the groups. No new coding themes emerged after 15 interviews for each group when we reached saturation with the data. We used the ATLAS.ti v.7 software computer program for qualitative data analysis, and we utilized a thematic content approach to analyze the interview transcripts [50, 51].

**Table 4:** Coding guideline.

FAMILY	CODE
Student Type	• High School • College ○ Science Major ○ Non-Science Major
Specific/Intended college & major/area of study	• Pre-Med • Biology • Biotechnology • Cell Bio Neuroscience
Courses taken in science and mathematics	• Identify Courses As Seen
Reason for Major (if College)	• General Interest • Family / Friend Influence • Trial & Error • Positive Experience • Interest in Research • Other
Program	• BCEP PROGRAM (C) • BCEP PROGRAM (R) • BCEP PROGRAM (S) • BCEP PROGRAM (B) • BCEP PROGRAM (SU) • Other
Program Duration & Type	• 120–400 Hours with Research • 120–400 Hours without Research • +400 Hours with Research • +400 Hours without Research
Interest in Scientific Research	• Yes • No
Prior Research Experience	• Yes ○ Positive ○ Negative ○ Indifferent • No
Career Intention Prior to Program	• Research Related • Non-Research Related
Reason for Participation	• Peers • Financial (i.e. stipend) • Opportunity to gain experience • Good on Resume • Interest in Research / Curiosity • Other
Career Intentions Post Program Changed	• Yes–Career Intentions has changed • No–Career Intentions has not changed
Length of Program–Factor in Influencing Career Intention	Students felt program was: • Too Long–Made up their mind in less time than what they were provided • Too Short–Needed more time to make decision on whether they liked research • Just Right–Perfect amount of time to make decision on whether they wanted to pursue a career in the sciences
Family Influence on Research Related Careers	• Yes ○ Positive ○ Negative • No
Friends / Peers Influence on Research Related Careers	• Yes ○ Positive ○ Negative • No
Mentor / Graduate Student Influence on Research Related Careers	• Yes ○ Positive ○ Negative • No
Self-Reported Efficacy / Competency	• Increased • Decreased • Same
Recommend Program to Friends	• Yes • No • Only Under Certain Conditions
Program Benefits	• Exposure to Research • Networking Skills • Job Skills • Build Friendships / Support System • Resume Builder • Helped in School / Grades • Increased Interest in Research • Other
Suggested Program Improvement	• Better Organization • Better Navigation • Better Guidance (i.e. More Lectures) • Better Communication (i.e. w/ Mentor) • Other
Perceived Deterrents to Careers in Scientific Research	• Stressful–i.e. Competition for Grants • Non-Lucrative • Instability of Projects • Perceived Lack of Human Contact • Lack of Results in Projects • Lack of Recognition • Other
Ultimate Career Plans	• Research Related • Non-Research Related • Medicine • Other
Year in high school or college	• 1st • 2nd • 3rd • 4th
If in high school, intended college & major/area of study	• Identify As Seen

We continually strove to remain objective and avoid perpetuation of bias in the themes identified through interview analysis. When we made inferences, all efforts were made to utilize existing clues from the participants’ responses. Even then, all efforts were made to clarify the true meaning behind a specific response. Lastly, in order to analyze the responses we developed a set of codes, which we used to analyze the unstructured data. (Table 4: Coding Guideline) We developed the codes based on key variables of interest in the literature, as well as from careful reading and rereading of transcripts for themes. The coding of responses followed a rigorous process; however, we did not use multiple coders to check inter-coder reliability.

## Results

Main qualitative themes that emerged from the data included:

1. interest and experiential learning in the context of specific professional and career goals motivates students from underrepresented groups to participate in BCEPs with research experience;
2. duration of research experiences in BCEPs influences research self-efficacy beliefs, but not research career intent;
3. BCEPs that offer research experience, family and mentors influence research career intent of underrepresented students; and
4. participants of BCEPs with long-duration research experience identify deterrents to research career intent.

### Interest and opportunity for experiential learning in the context of specific professional and career goals motivates students from underrepresented groups to participate in BCEPs with research experience

**Fig 1:**
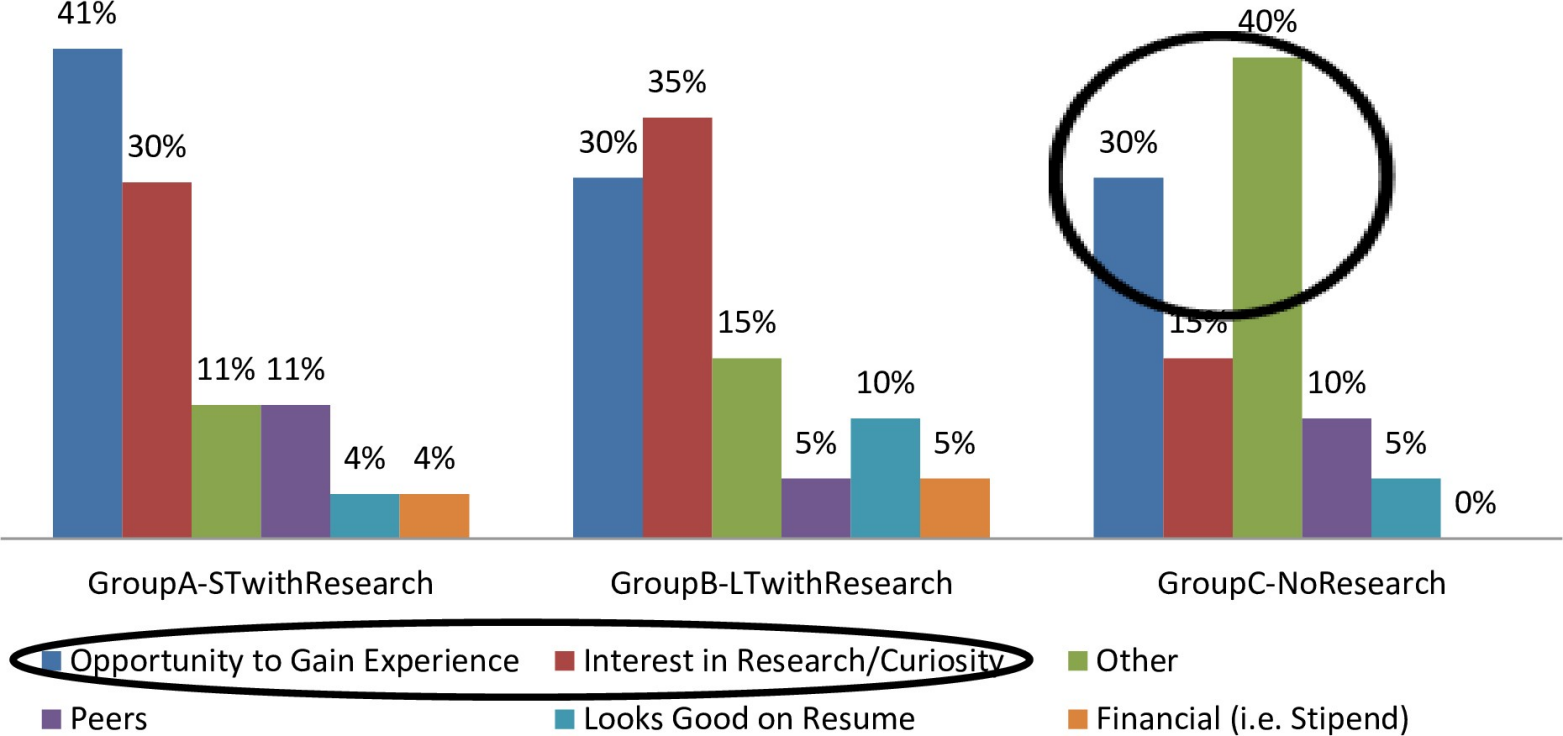
Motivations for participating in Biomedical Career Enrichment Programs.

Most students in research-based BCEPs—Group A (71%) and Group B (65%)—cited two main specific intrinsic motivators for participation in a structured enrichment program with research experience. These were an “opportunity to gain experience” and “an interest or curiosity in research.” The desire to make parents proud of their achievements also served as an intrinsic motivator for some of these students. However, a large percentage (40%) of participants in Group C (the No research BCEP), responded with non-specific motivators for participating in a BCEP, rather than an opportunity to gain experience or an interest in research (Fig 1).

Students in Group A (BCEP with short-duration research experience) articulated motivation for participating in the research-based BCEP in the context of future professional and career goals. One student explained her participation in a research-based BCEP in the context of her long-term goals to pursue graduate school:

“I wanted, for me, getting exposed in the medical field and science field—it doesn’t hurt going into it. And if you’re eligible then why not. And it is also in the city where I live and I’m planning on going into … a bigger or higher school and actually telling people that I worked for the program and I did cancer research that is appealing. Since I want to be someone in the medical field, having exposure to research, which I’m probably going to do or techniques that I will probably be performing on my way to MD or Ph.D..” (Group A)

Another student in Group A indicated research as a step that needed to be taken to pursue the long-term goal of teaching:

“… I always thought…research findings were interesting. Never really in the research process. And I would say, not until really recently. Because I guess it is really a means to an end. Like, I like seeing all these new things that get found or what not, and seeing how people find all these things, but it has never been “I really want to do research”, it is more of “I want to do that” but in order to do “that” you need to do research. Or…I have always wanted to teach and in a sense I would love to teach at some upper level, and so for you to do that (teach), you need a Ph.D. and to get a Ph.D., you need to do research. So it’s more of research is a couple of steps I’m taking in order to get there versus I really enjoy research and so I want to do it. I mean I don’t hate it. I still think it is interesting. It is just not my main love…”

Students in Group B (the BCEP with long-duration research experience) further emphasized the opportunity for experiential learning and interest in research to meet future career goals as their main motivators for participating in the program. One student in Group B said:

“I feel like the long-term programs would allow me to learn so much more. You know, even though, in the future I want to be a physician in public health, I feel like this experience has definitely taught me so much that I feel like if I was in a program for like three months I would not have learned so much. For example, there are few courses I haven’t taken yet and working with my mentor, he is allowing me to think critically by asking me questions about things. It is not about just learning information and memorizing it. You have to learn the process behind it. And that is something that I have been able to learn in the lab, but I can also use in other areas, even in my classes and in the future.”

Overall, several interview participants in Group B talked in detail about the motivation for research experience in the context of interest and experiential learning for career-decision making. Participants reported using research experience to test the possibility of pursuing an MD/Ph.D. degree and career as a science researcher or physician investigator. One student in Group B reported:

“I first started taking an interest in research in my freshmen year. I took a cancer seminar and that is when I started looking more into cancer research. And when the opportunity came for the [BCEP] program that is when I asked myself if this is something I should do or try out. I wasn’t sure if I wanted to do pre-MED/MD or MD/Ph.D.. So I thought it would be a good experience to try out and do research.”

Another participant in Group B, initially considering a career as a physician/researcher, elaborated:

“Because initially when I came into college I thought I wanted to be an MD/Ph.D. so I wanted to make sure I got a good experience in the lab to see if that was something I wanted to pursue. Resume building was a factor but not the major one.”

The desire to make parents proud of their achievements also served as an intrinsic motivator for some students. A student in Group A explained:

“My motivation for the science and [research] exposure is my Mom and Dad’s sacrifice in getting me to where I am. They don’t say it, but it’s implied. They don’t sit with me and say they are working hard so I can do this, this, and this. They’re working hard so that I can have the opportunities to seek what I want because they didn’t have that opportunity. I’m taking advantage of what they couldn’t have. This whole idea of an American Dream, basically I’m their American Dream. They are working hard so that I can be the best I can. So working here was an influence, they feel proud that I got into this program. It’s rewarding for me and them. So every single time, anything that is educational for my career and future, I always think about them. Not that if I’m going to make them happy, but is it worthy of the work they have done.”

Another student in Group B (BCEP with long duration research experience) elaborated:

“My parents were not born here. My Dad left me and so he doesn’t have a say in this. But my Mom, she just wants me to do something with myself. She really doesn’t understand, like every time I went to work and I would come back, I would try to explain to her what I was doing, but because she doesn’t know English and stuff, it’s kind of hard. I would try and translate but she doesn’t understand. It made me feel bad that she couldn’t understand me because I want to be able to explain what I’m doing so she can be proud of me but I already know she is. It’s fine.”

When we asked participants of Group C (the BCEP with no research experience) about their motivations for participating in the program, they responded with non-specific reasons “other” than an opportunity to gain experience or an interest in research. These reasons varied and included: following in a sibling’s footsteps, the flexibility and proximity of the program, the ease of the application process, a second-choice option because the student could not get into another program, or that the participant felt it was required of them because it helped fulfill one of their graduation requirements.

One participant in Group C said:

“I chose it because it was closer to home, I also noticed that they have the courses that I’m currently taking and that’s what I need for college, so that was like a perfect placement for, compared to the other programs that would show you around the hospital like that. I wanted more of an academic exposure.”

### Duration of research experience in BCEP influences self-efficacy beliefs about research skills

**Fig 2:**
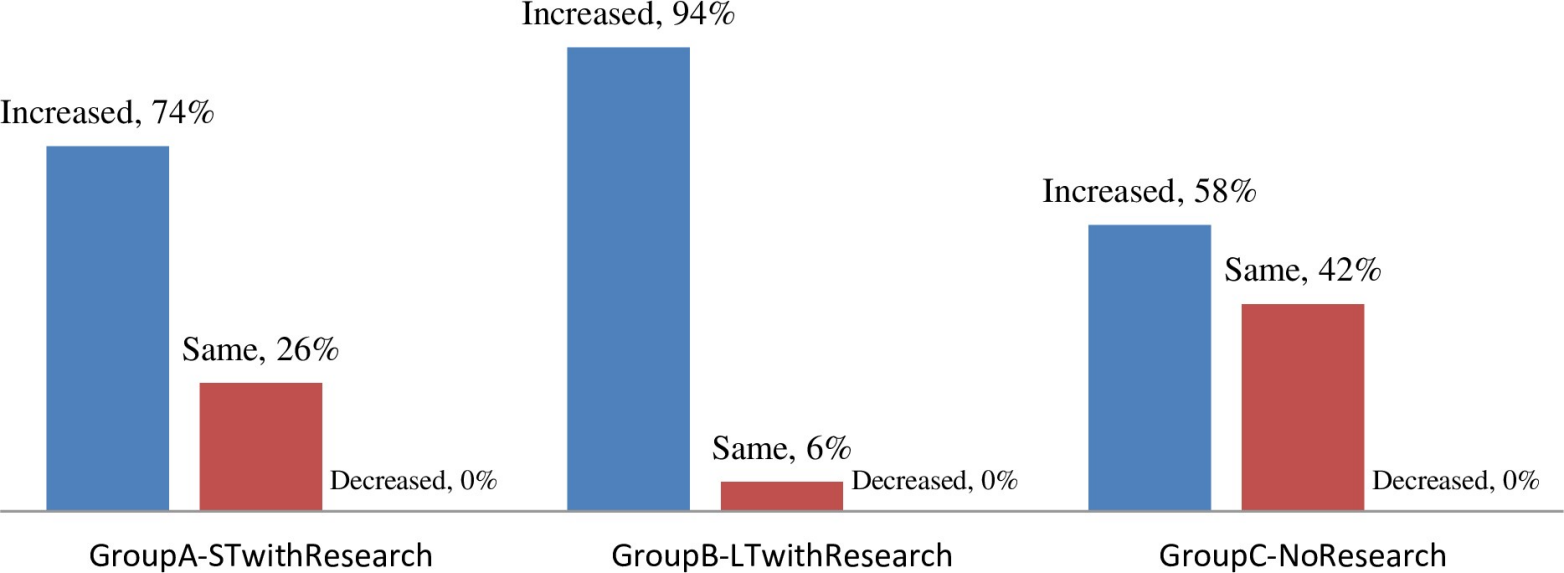
Self-reported efficacy change (pre-post) in students in Group A, B and C.

Participants were asked four different questions to assess how confident they felt in different areas (Table 3). Participants showed a difference in sense of pre-program to post-program self-reported efficacy. Students who had a longer exposure to research reported feeling more competent in their ability (1) to determine what is or what is not valid scientific evidence, (2) to interpret data tables and graphs, (3) to pose questions that can be addressed by collecting and evaluating scientific evidence and (4) to write a report using scientific data as evidence. It was clear as shown below in Fig 2, that longer duration resulted in better efficacy as seen through the results of “self-reported efficacy” where Group B (94% noted increase) exhibited more of an impact with self-reported efficacy than Group A (74%) or Group C (58%).

What is important to note is that the difference between the number of students who self-reported increased efficacy/competency (based on four questions listed above) and those who reported no changes is largest in the group with long-term research experience. While students in Group C with no research experience also self-reported some increase in efficacy/competency, this increase could be attributed to the science courses in which they participated.

Students who participated in Group A talked about increase in their confidence levels after participating in research. One student stated:

“I feel like it [competency] as definitely increased because I mean because you don’t really have confidence in something you have never really experienced in before but now I know that I can go in and do research.”

Students in Group A talked about an increase in their ability to evaluate scientific evidence. A participant explained:

“Papers get me a while to get through, but I feel more comfortable looking at them now. It’s not just hieroglyphics…it’s more familiar and less intimidating.”

Most students (94% of the students) who had a longer exposure to research (Group B–Long Duration) self-reported gains in confidence levels. One student reported:

“I’m still working on this but it’s improving. On a scale from 1–10, I’m at a 6–7. I’m more confident than when I went in.”

Self-reported gains in research self-efficacy skills observed in participants of Group A continued in those students who participated in Group B. One student from Group B expressed increased confidence in reading scientific literature and enhanced communication skills in discussing research because of participating in the BCEP:

“I think it is definitely a lot better. Like now I know that it is okay to make mistakes that it just happens all the times after being here for a while…and how to think about reading papers because we have Journal club every month…and talking about my research and I’m a lot more comfortable with it.”

Students in Group C (BCEPs with no research experience) as expected did not report similar gains in research skills self-efficacy following participation in the BCEP as compared to those reported by students in Groups A and B. They attributed some gains in scientific research skills to the academic courses they took because of participating in the BCEP. One student in Group C said:

“Through the program, I don’t think so but through the courses that they recommended, yes. For example, the second Expository writing class that focuses on Science & Research. I know it helped us to learn to read scientific articles and things like that in order to write a paper like that. I would have never known about this course had it not been for [Program].”

### Duration of research experience in a BCEP does not influence outcome expectations and research career intent

Students discussed whether the duration of their experience in their BCEP was adequate in meeting their professional goals for participation and if exposure to laboratory research specifically in the BCEP had an influence on their career choice. Students in both Groups A and B (BCEP with research) self-reported that the duration of the program was “Just Right” for their professional goals. Participants self-reported that the time spent in the BCEP provided valuable experience in scientific research and exposure to a career in research. In Groups A and B, the majority of students felt the program duration was “just right”. (Group A– 50%, Group B– 86%).

To further probe what “just right” meant to the students, they were asked, “Would you have reached your choice for your ultimate career goal if given half of the time in the program?” For most students, BCEPs with long-term research experience was important for understanding the research process and getting an adequate learning experience but not for ultimately affecting career choice. The following quotes are from the responses of participants of Group B:

“I think it was the perfect time. Because I was working full time so that I was getting an idea of what it would be like if I did go to graduate school. Then, it was enough time to get that idea. I think half the time is too short because first of all you cannot do work in five weeks and you cannot get the feel that the project is truly yours. We also attended seminars weekly and I think five weeks is too short to really get adapted to and understand your research.” (Student, Group B)

“Even during the presentation this summer, and this summer I think I got the whole feel back of it, the whole feel again, like I really liked doing research this summer but I think the first summer is like the honeymoon stage. So I think you need the two years because the first summer is the honeymoon period because you love everything about it and everyone is so nice and the second year, they expect more from you, they expect you to know which primary antibody goes to axel, etc. and you need to know these things.” (Student, Group B)

When the effect of the research experience on career intent was examined, a majority of the participants in Group A reported that the length of research experience did not change their original career choice from when they started the program. Sixty percent of the students in Group A said the experience (BCEP with short duration research) did not change their original choice and either persuaded them away from research or reinforced their initial career choice. In Group B (BCEP with long duration research), there was almost an even split in students wanting to go into research and those stating that research was not for them.

Students were further probed to understand why the research experience deterred them from research careers or reinforced their original career intent. Two students in Group A explained why they were deterred from a research career:

“…I think it kind of helped me realize, like, I do want to do Oncology and…I don’t think I want to do research as a career but it was good looking into it…”

“…I know that I had an interest in public health. Now I know I will not be into the Epidemiology part of public health which deals with more…science stuff–like we are doing here. Now I know other fields of public health [non-research] would better for me…”

As students spent more time in research environments (Group B), they reported feeling intimidated due to the lack of role models of same gender/ethnicity.

“…Prior to this program, I wanted to be a physician and a public health advocate. I was considering a career in research. I think the program allowed me to see the work environment. I saw that in research there are so many men in the labs and also race plays a huge role in it. Yes it would be great if I was the first black person in the lab and I’m getting results but then it is also intimidating because there are also people always looking at you to see what mistakes you are making. For me, being an undergrad, I think that is something I’m reminded of on a daily basis. That is another reason why I am just backing away from it.”

Participants also elaborated on the lack of interaction with people and isolating nature of science and its influence on their intent to not pursue a research career.

“So I guess before, I didn’t really know what research was about so I was probably more open to the option of research as a career. I didn’t think I would like it. But I think now, I have gotten a sense of it and I think I can say more firmly that I probably wouldn’t enjoy research. I really admire researchers and I think what they do is great. I did really enjoy my experience in the lab over the summer and I’m actually continuing over the year. But for a career, I would want one with more interaction I guess with the public instead of staying in the lab working by myself all day. That [interaction with public] is something I would really enjoy.

The current realities of the academic labor market with lack of funding and its impact on laboratory research negatively influenced the research career intent of students in the BCEP with long-duration research experience. As one Group B student said:

“…it did influence me a little bit because it is quite intimidating when you see labs and people losing their jobs or getting shut down. It is like, wow, I don’t want to be in a place where I cannot get another job, and I want financial security.”

Another student in Group B said:

“I know that a Ph.D. is awesome but it depends on what you go into because a Ph.D. can be good in some places but not in all places. I think they taught me that well enough so it helped me decide what to do. They (mentors and graduate students) were very realistic. Even there was a recent scientist that just entered the lab and she has been talking to me and she has been realistic about what the opportunities are for Ph.D. these days and I think, I knew those already, but its either industry or research and pure research depends on grants and grants come from government and what I learned is that NIH has decreased a lot of grants so it is really hard and more competitive. *“*

### Family modulated participants’ intent to pursue research career in this study

When examining the role of family in career decisions, participants self-reported that most family members in all three groups were supportive of the career choices of the student, as long as that student was happy. In Group A, 47% of the students self-reported that family positively influenced their pursuing research-related careers, 18% reported negative influence of family on research-related careers, and 35% of the respondents reported no overall influence. Interestingly, among participants of Group B, the role of family as a positive influence on research-related careers decreased to 13%; the majority reported family as having no influence (60%), with 27% noting a negative influence—the highest when compared to all three groups. Majority (56%) of the students in Group C self-reported that the family had little to “no influence” on their career choice.

To further explore the influence of family on research-career choice, the participants in all groups were asked to elaborate if family/peer, teacher, instructor, or the program they were participating in had encouraged or discouraged them for pursuing a career in scientific research. Surprisingly, a few students with parents or other family members already in the research field were discouraged from going into research. Whether this was due to lack of stability or profitability is unclear. When asked if students felt discouraged by these factors, the majority reported family opinions mattered, but they did not influence their ultimate career choice.

“And in terms of discouraging, surprisingly my Mom. Because she herself got a Ph.D. and then did research and then she did not get a very good experience out of it, and she in general thinks that it is a lot of work for very little reward, and so she has not, I would not say, full on discouraged but she has not been like she is going like “Go for it!” (Student, Group A)

“My oldest brother. I’m very close with him–more than anyone ever. He is a patent attorney. He is a Rutgers alumnus, he was an engineer but now he is a lawyer. He is supportive of me doing it [research] while I am in school. But as a career he thinks it should be a Plan B.”(Student, Group A)

“Well, technically, my Mom always wanted me to be a MD or a dentist and she always saw research as a very big step. That is likely why I am not going into research. For some weird reason, I don’t think she sees research as being very glamorous as much as an MD. I think that is possibly why I pursued an MD as a career, instead of just doing a Ph.D. or something like that.” (Student, Group C)

### Peer influence is not linked to research career decisions of BCEP participants

Most participants reported friends and peers had no influence on their career choice: Group A (50%); Group B (57%); and Group C (63%). Friends and peers were often noted as being a support system more than anything else, as many of the participants and their network of friends were in similar majors, programs, and career paths.

“My peers are in the same boat as I am. We kind of just talk about research. None of us really want to be in research later on” (Student, Group B)

“The peers that don’t know my research; they’re like “oh that’s cool.” Both of them have been positive I think. I have a couple of peers who have been doing research and I think it is really cool how you can talk about the things we are doing in lab and then you have other people who are like “what are you talking about?…I think they have both been very positive. No one has ever discouraged me or said that’s bad”. (Student, Group B)

### Mentors influence research-career decisions for participants

The mentors and graduate students served as a significant source of influence on career choices of students in both Group A and Group B. Mentors and graduate students were reported to have had the most negative influence on Group A (33%) in the short-term research experience. Whereas the opposite was observed in Group B (longer-term research), where 53% of the participants described the mentors and graduate students as having had the most positive influence.

Students in each group were further asked to describe the processes through which mentors positively or negatively influenced their career decisions. One student in Group A articulated being inspired by their mentor to contribute to important research in the biomedical field and to increase gender diversity in the field.

“My mentor really influenced me. I want to do research. When I was in the lab, I wasn’t passionate about esophageal cancer but I kind of want to go into that because of my mentor—who told me that it was one of most commonly occurring cancers that has practically no treatment available. You pretty much die instantly. Not a lot of people go into this specific field. So I feel like I would want to go into this field just to contribute. And plus he said not a lot of women go into research so I feel I should go just to prove them wrong.” (Student Group B)

On the other hand, graduate students serving as mentors shared their own negative perceptions of their training and discouraged the research-career intent of some participants.

“In talking to some people who are getting their Ph.D. or have already gotten it in the science field …they all complain about grad school and how awful it is and how poor you are and how long it is and stuff and how much work it is and stuff and that kind of is disturbing but when you see how successful they are now it kind of, you think it’s worth it even though it is so long being a poor college student. It definitely made me think twice about it. Just because maybe the length of the schooling is what is holding me back from it but I think my other feelings about wanting to get a Ph.D. override some of these feelings.” (Student Group A)

Another student from Group B described how mentors spoke of negative experiences when giving advice to the participants.

“I’ve spoken to a few of my mentors and grad students and I don’t know why but the majority of people I spoke to would not encourage their children to do research. Maybe because a majority of the people I have spoken to are MD/Ph.D. and male.”

### Specific deterrents to research-career intent described by the BCEP participants from underrepresented groups

**Fig 3:**
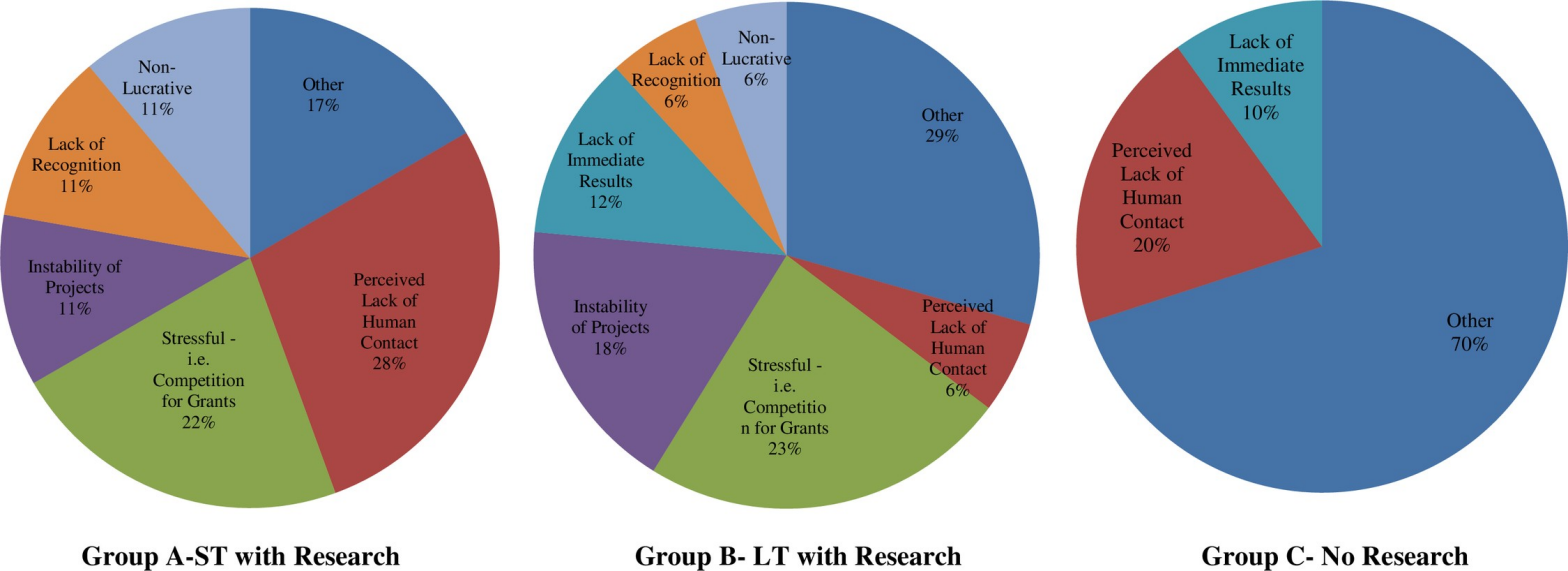
Perceived deterrents to careers in research.

The study revealed an interesting and unexpected theme of loneliness as a perceived deterrent, especially among Group A, which discouraged many of the participants from further pursuing research careers. Fig 3 depicts the perceived deterrents of careers in research sciences. In Group A, many students perceived research as a lonely career in that there was a lack of patient contact. However, for those students exposed to longer research experiences (Group B), this was less of a problem. For Group B students, other reasons, such as stress of competition (e.g. grants), the instability of projects, and lack of immediate results were more important than the lack of human contact. The majority of Group C (70%) gave “other” reasons than the major deterrents identified in Group A and Group B. These reasons included such characteristics as “difficulty” of research and perceived “intimidation” about that level of difficulty. Other reasons also included negative perceptions of research being redundant and uninteresting.

In Group A, a little over a quarter of the respondents (28%) spoke about the fact that many perceived research as a lonely career. They felt that there was very little patient or human contact.

“I don’t know, based on my personality, I don’t see myself working in the lab as my job, I prefer to work in a hospital, as more of a hands-on kind of thing, interact with patients, just more of a personal level. I feel like working in a hospital or a clinic, you gain more of that personal interaction than say working in a lab.” (Student, Group A)

“Research, no. I really did enjoy my time here. But honestly, the grant situation, like my lab is closing down this summer. And last summer the number of people were cut in half. To me it doesn’t seem like a very stable field from what I have experienced. I just want that stability for when I get older and I have a career. (Student, Group A).

Students in Group B (41%) also articulated the stress of competition (e.g. for grants), the instability of projects, and the lack of immediate results as greater deterrents than the lack of human contact. Apparently the longer duration of research exposed participants to more of the negative realities of academic research.

“Prior to going into the program I was interested; I just wanted to get my feet wet and just to see how the environment would be. I just feel like it is a lot to learn, it’s a lot to take on and on a daily basis, whenever we are doing different experiments, if you do five experiments one day or for a few months, you may not get results, so I think that aspect of it is what draws me away from it…the aspect of constantly not knowing if you’re going to get results is what sets me back.” (Student, Group B)

The majority of Group C (70%) offered “other” random deterrents rather than the major themes identified in Group A and Group B.

“Well, just in general, I like working with people. But also because, I don’t know, like, with lab there is a lot of tedious work that I don’t like dealing with.” (Student, Group C)

## Discussion

Empirical data on why and how students from URG choose non-course-related scientific research experience is lacking. Our exploratory study with students from underrepresented groups highlights their motivations for pursuing research-based versus non-research-based BCEPs. It also provides a more nuanced picture of the participants’ experiences in the biomedical research laboratories and how the experiences influenced their career interests. Students initially participating in research-based BCEPs identified “opportunity to gain experience” and “interest or curiosity in research” as the main motivator in the context of their professional and career goals. In contrast, students who chose BCEPs with no research experience reported a variety of other non-specific contextual motivators for participation unrelated to skill development and/or interest.

In a study by Smith et al. [52] to explore the reasons why individuals engage in science research, latent profile analyses of 1,052 undergraduate students showed “intrinsic” and “extrinsic” motivators influenced science-class experience, identity as a scientist, and future intentions to pursue scientific research. Examples of intrinsic motivators are “satisfaction from intellectual challenge, scientific processes itself, or the discovery of new information,” and extrinsic motivators are “academic recognition, graduate school admission, and potential for published journal article.” Our study supports and extends the study by Smith et al. [52] and documents that interest in research and opportunity for experiential learning within the context of biomedical career goals are significant motivators for students pursuing research-based BCEPs. Future studies should focus on dissecting what influences a student in a research-based BCEP to ultimately pursue a research career and determining if introducing a BCEP with research at a certain academic level makes a difference, especially for students from underrepresented groups for whom these programs are designed.

Students participating in longer duration of research experience in a BCEP, self-reported gains in evaluating the validity of scientific research as well as hypothesis, interpretation, analysis, and communication of scientific data. Thus, students with no prior research experience might particularly benefit from a BCEP with research as the experiential learning enhances analytical skills useful in broader STEM related careers. These observations add to prior studies showing gains in research skills in participants of summer research experiences [38, 39].

Interestingly, neither research experience in a BCEP nor the duration of the research experience persuaded participants to pursue a research career. The majority of the students in each group self-reported either being persuaded away from research or reinforcing their initial career intent to pursue a medical degree. Longer exposure to a research program did not necessarily result in different outcomes or change in career intent. While prior studies have examined gains in research skills [8, 39], graduation rates [5], and intentions to pursue a graduate degree [18], no studies have examined the research-career intentions of participants in BCEPs. Our results extend prior research and have implications for policy-level decisions, specifically in articulating a strategy for effective program design to establish more short-duration research programs. Such short-term research programs would reach more minorities and women, increasing the number of underrepresented students going into a career in a STEM field.

In addition to duration of research experience, this study also looked at underrepresented students’ support systems in influencing career decisions (i.e., role of family, peers, and mentors). Research in this area has been equivocal. Consistent with the study done by Layton et al. [31], while students in our study verbally reported that the opinion of their family carried little weight on their final career choice, there were implied influences present. These implied influences might have manipulated the student’s ultimate perception and decision towards a research career. Future research could examine factors such as the level of education and prior research-related experiences of parents and siblings and their possible link to levels of family support for careers in scientific research. These findings could help direct efforts to reduce negative perceptions articulated by study participants about careers in research.

For many underrepresented students, communicating with family members about career opportunities was difficult due to language barriers and/or limited parental exposure in the area of interest. Friends and peers, for these students, acted as the next best intimate source of advice and guidance, shifting the social/cultural capital available to them to make informed career decisions. Despite pessimism experienced in informal conversations with friends and peers describing research as being “boring” or “cumbersome,” participants reported that friends and peers were generally supportive and encouraging of a research-career intent.

The study revealed an unexpected finding of perceived deterrents to science research careers and retention in STEM/scientific research. Although there is some data on barriers to initial participation in science research programs, there is a paucity of data on program processes that discourage participants’ retention in scientific research careers. Our study suggests that longer durations of research in BCEPs expose students early on to the adversities of US biomedical research enterprise. These include the decrease in federal research funding, hyper competition, and fewer academic job opportunities as articulated in the 2012 report by the NIH Biomedical Research Workforce Working Group led by Shirley Tilghman [53]. The findings of this study reflect the challenges currently plaguing the US biomedical research enterprise at the graduate student, post-doctoral, and workforce level. However, it is interesting to observe how the trickledown effect of professional discouragement can impact research-career intent in participants of BCEPs.

While prior studies document powerful influence of mentors on research career intent of students [23, 25, 34, 54], mentors and graduate students served as a surprisingly discouraging source for advice and guidance for students in our study. The mentors were either senior graduate students or post-doctoral fellows in the laboratories of the principal investigator and were not selected to be of the same gender and/or racial/ethnic background as the mentee in our study. The students from Groups A and B spent the majority of the time with their mentors who closely supervised the students in short- as well as long-term research based BCEPs. Interactions with mentors and graduate students were often difficult for participants in the beginning. Responses suggested that the student and mentor did not build a solid relationship that would allow students to feel comfortable in asking questions and becoming fully engaged in the project, even though the students spent 90–95% of their time in the laboratory interacting with the mentors. This could explain why students who were provided a shorter time in the program had more negative perceptions of research. This observation suggests the critical need for training mentors in skills needed for effective mentoring and developing positive mentor-mentee relationships in order to improve overall experience of trainees in BCEPs. Building a positive relationship with the mentor early on is crucial in a student’s perception of their experience, which ultimately might influence their career choices.

Our findings reveal that interest in research and opportunity for experiential learning serve as motivators for students who select research based BCEPs. However, the duration of research based BCEPs did not have an effect on the final career intent of the student. Surprisingly, many participants of research based BCEPs reported negative perceptions about research careers, including “lack of human contact”, “financial instability” and “grant funding”. In addition to participant perceptions, parents, mentors and other graduate students also contributed to participants’ decision to pursue a “non-research” career. These findings suggest several future avenues for research and increasing interest in biomedical research career pathways. First, it is critical to improve the scientific research-training environment with additional federal funding for research to reduce hyper-competition. Second, additional training and career development activities are needed at graduate and post-doctoral levels to improve career outcomes and satisfaction of biomedical trainees who serve as mentors and role models for participants of BCEPs. Third, additional formal training is needed for those graduate students and postdoctoral fellows who serve as mentors for trainees in the BCEPs so they can effectively guide participating students. It is imperative to address the negative perceptions of students from underrepresented groups and encourage them to pursue research careers for increasing diversity in the biomedical research workforce.

## Limitations

Qualitative studies hold their own set of inherent limitations. The limitations of this study include (1) small sampling size; (2) establishing credibility from the responses; (3) avoiding social desirability, self-reporting, correspondence, and actor-observer biases.

Due to time restraints and scheduling conflicts, recruiting participants for the interview was difficult, and in turn, convenience sampling was at times unavoidable. Convenience sampling relies “…on data that is selected by those who provide it or those who observe it—information from individuals who chose to tell their stories” [55]. The number of participants in each group might not be fully representative of an entire population.

However, the objective of this study was to document experiences, and to better understand the *how* and *why* of the career decision-making processes of these BCEP participants, rather than produce generalizable results. In addition to the convenience sampling bias, it is also plausible that some respondents were susceptible to “Social Desirability Bias / Self-Reporting Bias”. These additional biases explain that self-reported responses contain certain limitations, including selective memory, attribution of positive events to oneself while negative events to someone or something outside oneself, and embellishment or exaggeration of events. We took all of these factors into consideration when conducting the overall analysis of results. Thus, any generalizations observed during the analysis of the coded data within this study will require a more extensive follow-up study to help authenticate the findings.

Credibility of responses is a central concern when conducting qualitative research. To build credibility, we made an effort to build a strong relationship with each respondent in order to elicit the most candid responses. We used member checks throughout the survey process to help establish the trustworthiness of responses and decrease incorrect interpretation of data. We did this by repeating and summarizing responses, then allowing each respondent to critically evaluate and determine the accuracy of their views, and further comment on these responses as needed.
